# Supplementary material for: Radiation Exposure Induced Blood–Brain Barrier Injury via Mitochondria‐Mediated Sterile Inflammation
Source: Adv Sci (Weinh). 2025 May 28;12(31):e02356. doi: 10.1002/advs.202502356 (PMC12376696; doi:10.1002/advs.202502356)
Supplement: Supplementary file 1 — Supporting Information [file ADVS-12-e02356-s001.docx]

**Supplementary Information**

**Supplementary Table 1. Primer sequences used for RT-qPCR analysis in this study.**

| **Primer** | **Sequence (5ʹ-3ʹ)** |
| --- | --- |
| GAPDH-F | CACCCACTCCTCCACCTTTGAC |
| GAPDH-R | GTCCACCACCCTGTTGCTGTAG |
| IFNE-F | GGAACTGTGTTGGTGCTGCTG |
| IFNE-R | TGGTAGACACTGCTGAATTGACAAG |
| IFNAR1-F | ACTCATTTACACCATTTCGCAAAGC |
| IFNAR1-R | ACCATCCAAAGCCCACATAACAC |
| IFNAR2-F | CCACTCCATTGTACCAACTCACTATAC |
| IFNAR2-R | GCACAGTTCTTAACCACCTTCAAATC |
| STAT1-F | ATGCTGGCACCAGAACGAATGAG |
| STAT1-R | TCACCACAACGGGCAGAGAGG |
| MX1-F | GCATCTCCAGCCACATCCCTTTG |
| MX1-R | TGGTGTCGCTCCGCTCCTTC |
| ISG15-F | TGGACAAATGCGACGAACCTCTG |
| ISG15-R | GCCCGCTCACTTGCTGCTTC |
| IRF7-F | AGAAGAGCCTGGTCCTGGTGAAG |
| IRF7-R | AGGCTGAGGCTGCTGCTATCC |
| IFIT3-F | TACGCCTGGGTCTACTATCACTTGG |
| IFIT3-R | CACTTCAGTTGTGTCCACCCTTCC |
| MT-ND1-F | CCACCTCTAGCCTAGCCGTTTA |
| MT-ND1-R | GGGTCATGATGGCAGGAGTAAT |

**Supplementary Table 2. Primary antibodies used for immunofluorescence in this study.**

| **Antibody** | **Vendor** | **Catalog #** | **Dilution** |
| --- | --- | --- | --- |
| Anti-VE-cadherin | Cell Signaling Technology | 2500S | 1:200 |
| Anti-GFAP | Cell Signaling Technology | 3670S | 1:200 |
| Anti-IBA1 | Proteintech Group | 66827-1-Ig | 1:200 |
| Anti-IBA1 | Proteintech Group | 26177-1-AP | 1:200 |
| Anti-CD31 | HUABIO | M1511-8 | 1:200 |
| Anti-p-STAT1 | Proteintech Group | 28977-1-AP | 1:200 |
| Anti-Ki67 | Abcam | ab243878 | 1:200 |
| Anti-53BP1 | Santa Cruz | sc-515841 | 1:100 |
| Anti-53BP1 | Abcam | ab175933 | 1:200 |
| Anti-dsDNA | Abcam | ab27156 | 1:200 |
| Anti-cGAS | Proteintech Group | 26416-1-AP | 1:200 |
| Anti-TOM20 | Proteintech Group | 11802-1-AP | 1:200 |

**Supplementary Table 3. Primary antibodies used for Western blot in this study.**

| **Antibody** | **Vendor** | **Catalog #** | **Dilution** |
| --- | --- | --- | --- |
| Anti-p-TBK1 | Cell Signaling Technology | 5483 | 1:2000 |
| Anti-TBK1 | Cell Signaling Technology | 3504 | 1:2000 |
| Anti-cGAS | Proteintech Group | 26416-1-AP | 1:2000 |
| Anti-STING | Proteintech Group | 19851-1-AP | 1:2000 |
| Anti-GAPDH | Proteintech Group | 60004-1-Ig | 1:2000 |

**Supplementary Table 4. Chemicals used in this study.**

| **Antibody** | **Vendor** | **Catalog #** | **Final concentration** |
| --- | --- | --- | --- |
| Hydrocortisone | Stemcell | 07925 | 1 μM |
| Crisaborole | MedChemExpress | HY-10978 | 10 μM |
| Abrocitinib | MedChemExpress | HY-107429 | 1 μM |
| SN-011 | MedChemExpress | HY-145010 | 1 μM |
| Coenzyme Q10 | MedChemExpress | HY-N0111 | 10 μM |
| BAI1 | MedChemExpress | HY-103269 | 2 μM |
| Idebenone | MedChemExpress | HY-N0303R | 20 μM |


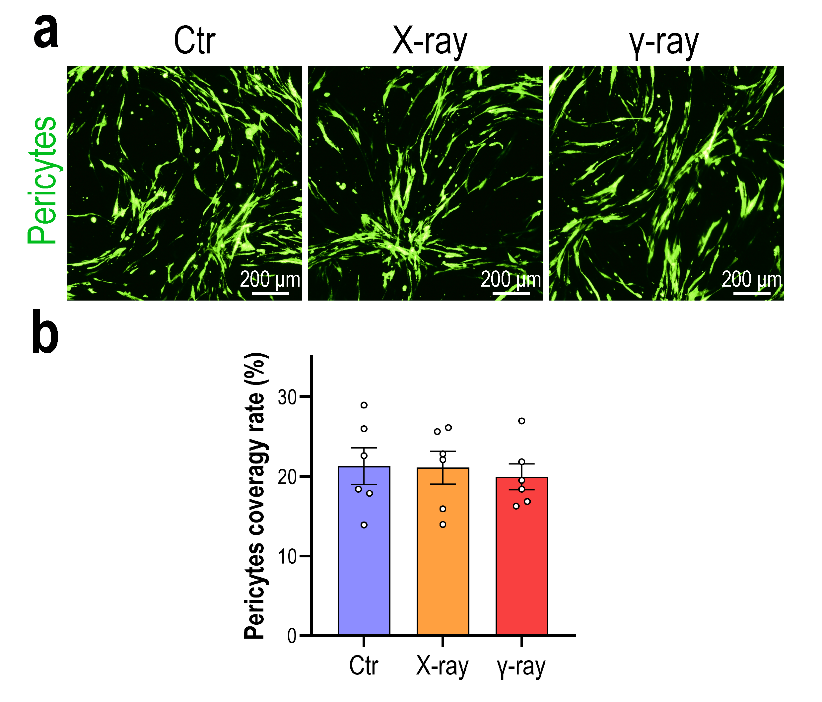


**Supplementary Figure 1. Fluorescent image showing pericytes on BBB MPS following radiation exposure. a**, Fluorescent image showing pericytes labelled with GFP on BBB MPS, 4 days following radiation exposure. **b**, Quantification of pericytes coverage rate based on **a** (*n* = 3). Two images were analyzed for each sample. Data are presented as the mean ± SEM and were analyzed using a one-way analysis of variance (ANOVA) followed by the Bonferroni post hoc test.


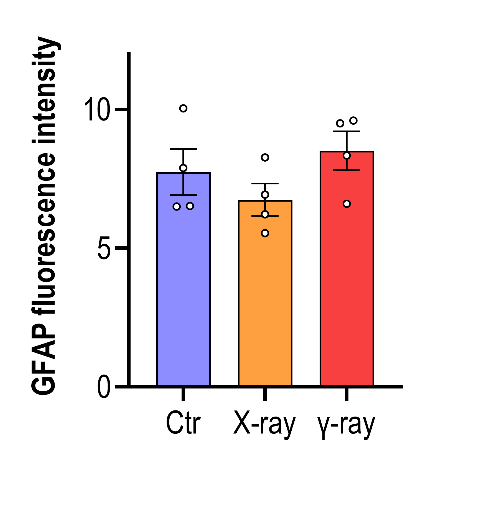


**Supplementary Figure 2. Quantification of GFAP fluorescence intensity for astrocytes on BBB MPS following radiation exposure.** Quantification of GFAP fluorescent intensity based on **Fig. 2f** (*n* = 4). Data are presented as the mean ± SEM and were analyzed using a one-way analysis of variance (ANOVA) followed by the Bonferroni post hoc test.


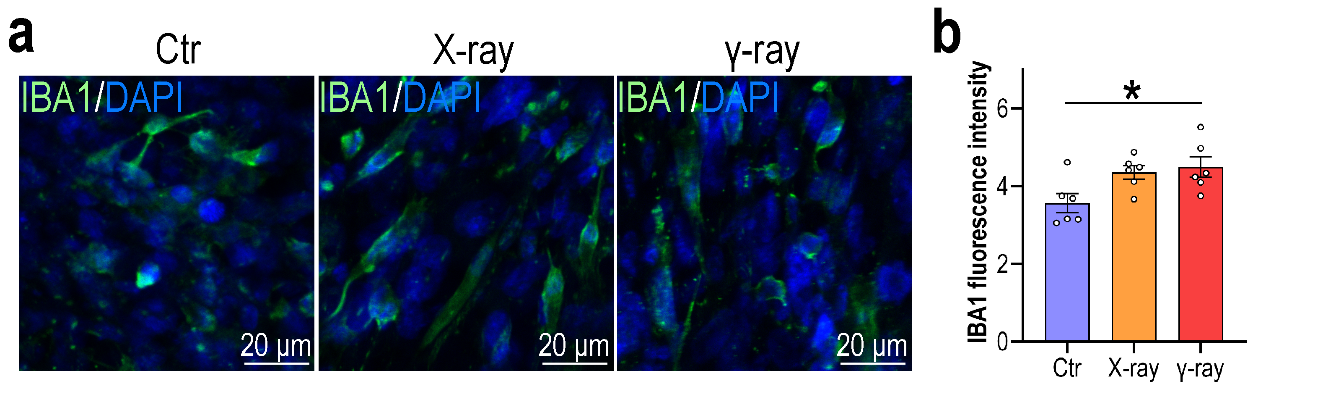


**Supplementary Figure 3. Immunofluorescent image showing microglia on BBB MPS following radiation exposure on BBB MPS. a**, Confocal micrographs showing microglia immunostained for IBA1 on the BBB MPS, 4 days after radiation exposure (*n* = 3). **b**, Quantification of IBA1 fluorescent intensity based on **a**. Two images were analyzed for each sample. Data are presented as the mean ± SEM and were analyzed using a one-way analysis of variance (ANOVA) followed by the Bonferroni post hoc test (*: *P* < 0.05).


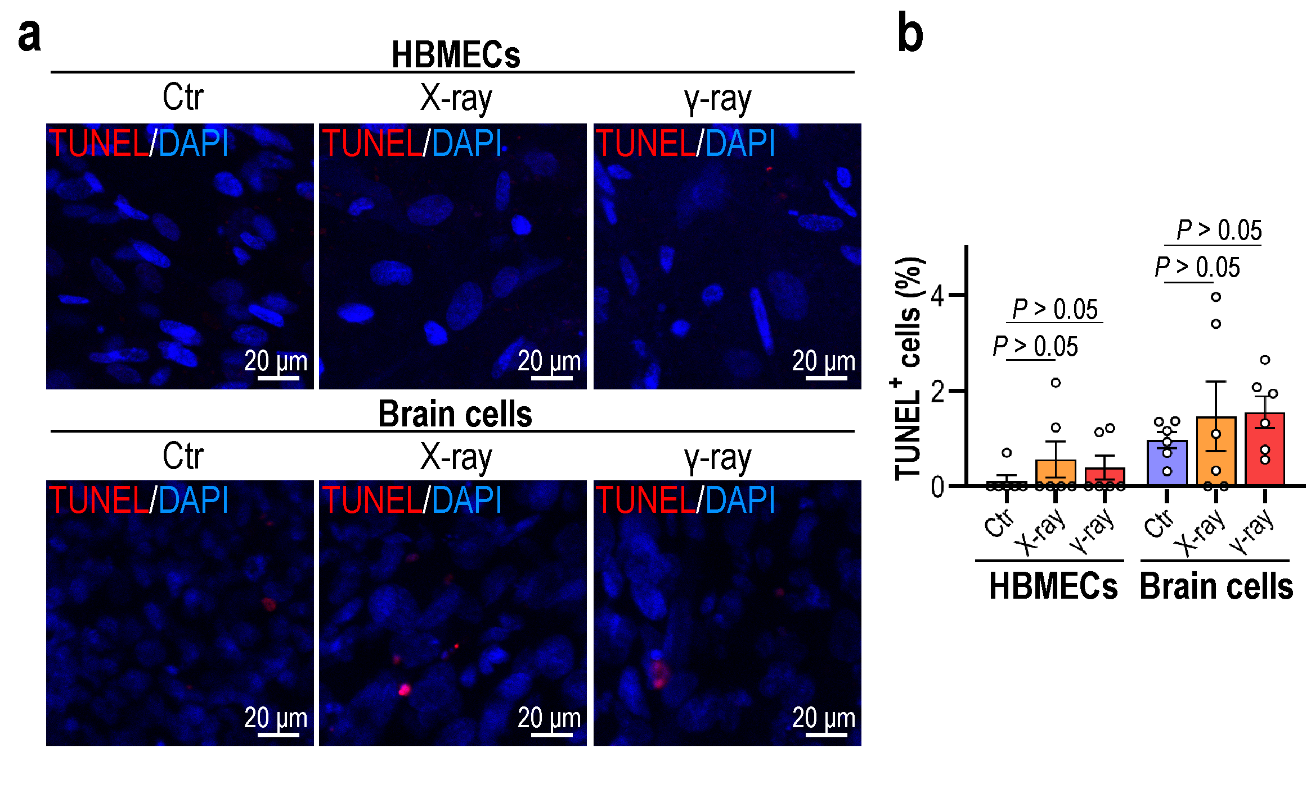


**Supplementary Figure 4. Apoptosis analysis for BBB MPS following radiation exposure. a**, Confocal micrographs showing cells stained by TUNEL kit on the BBB MPS, 4 days after radiation exposure (*n* = 3). **b**, Quantification of TUNEL+ cells based on **a**. Two images were analyzed for each sample. Data are presented as the mean ± SEM and were analyzed using a one-way analysis of variance (ANOVA) followed by the Bonferroni post hoc test.


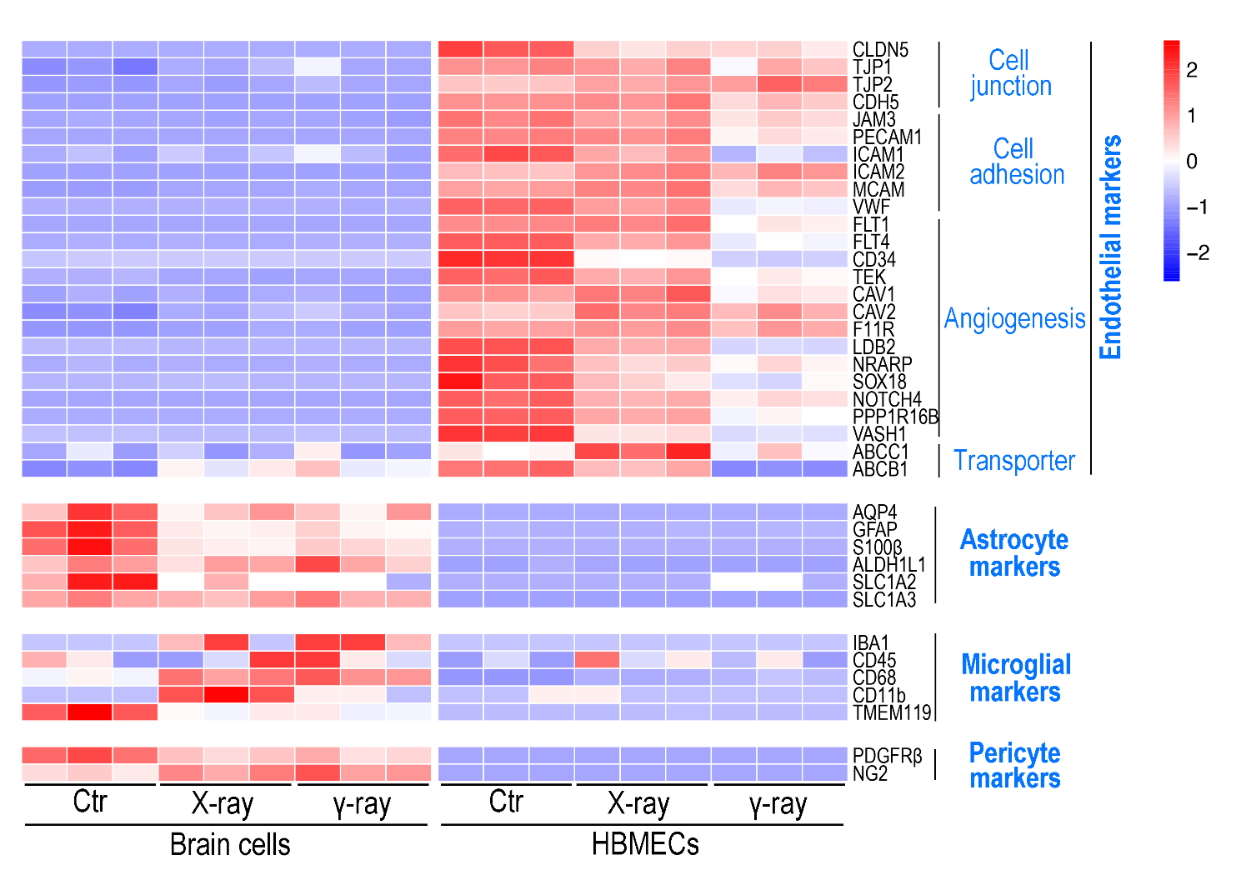


**Supplementary Figure 5. Transcriptomic analysis showing expression levels of cell-specific genes for BBB MPS following radiation exposure.** Heatmap showing the expression of cell-specific genes in brain endothelial cells, astrocytes, microglia and pericytes, 4 days after radiation exposure (*n* = 3). Genes differentially expressed with fold changes of > 2.0 and *P* < 0.05 are defined as differentially expressed genes (DEGs). *P* values were calculated using a two-sided, unpaired Student’s t-test with equal variance assumed.

**
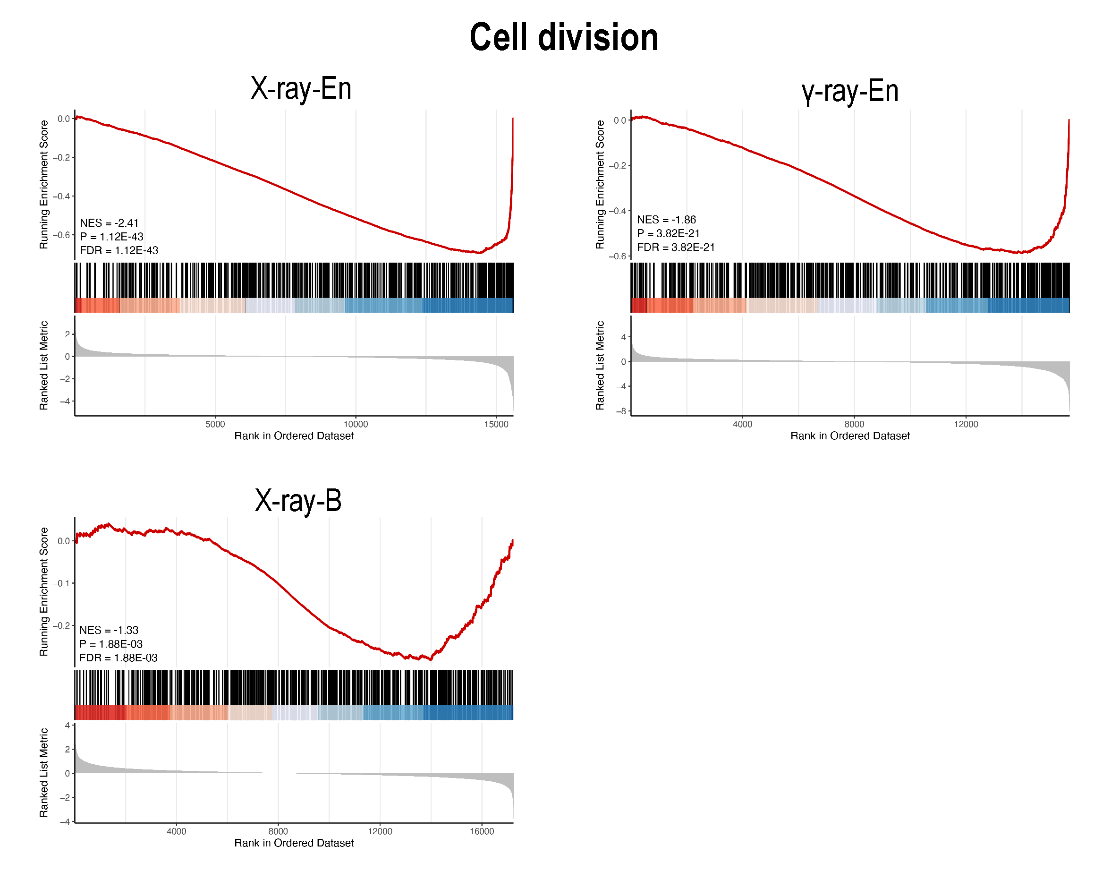
**

**Supplementary Figure 6. GSEA analysis of cell division process for brain endothelial cells and brain cells on the BBB MPS following radiation exposure. a**, GSEA analysis reveals correlation between radiation exposure and genes involved in cell division in brain endothelial cells. **b**, GSEA analysis reveals correlation between radiation exposure and genes involved in cell division in brain cells. **a-b**, NES, normalized enrichment score. FDR, false discovery rate. Gene sets were considered significant when *P* < 0.05 and FDR < 0.25.


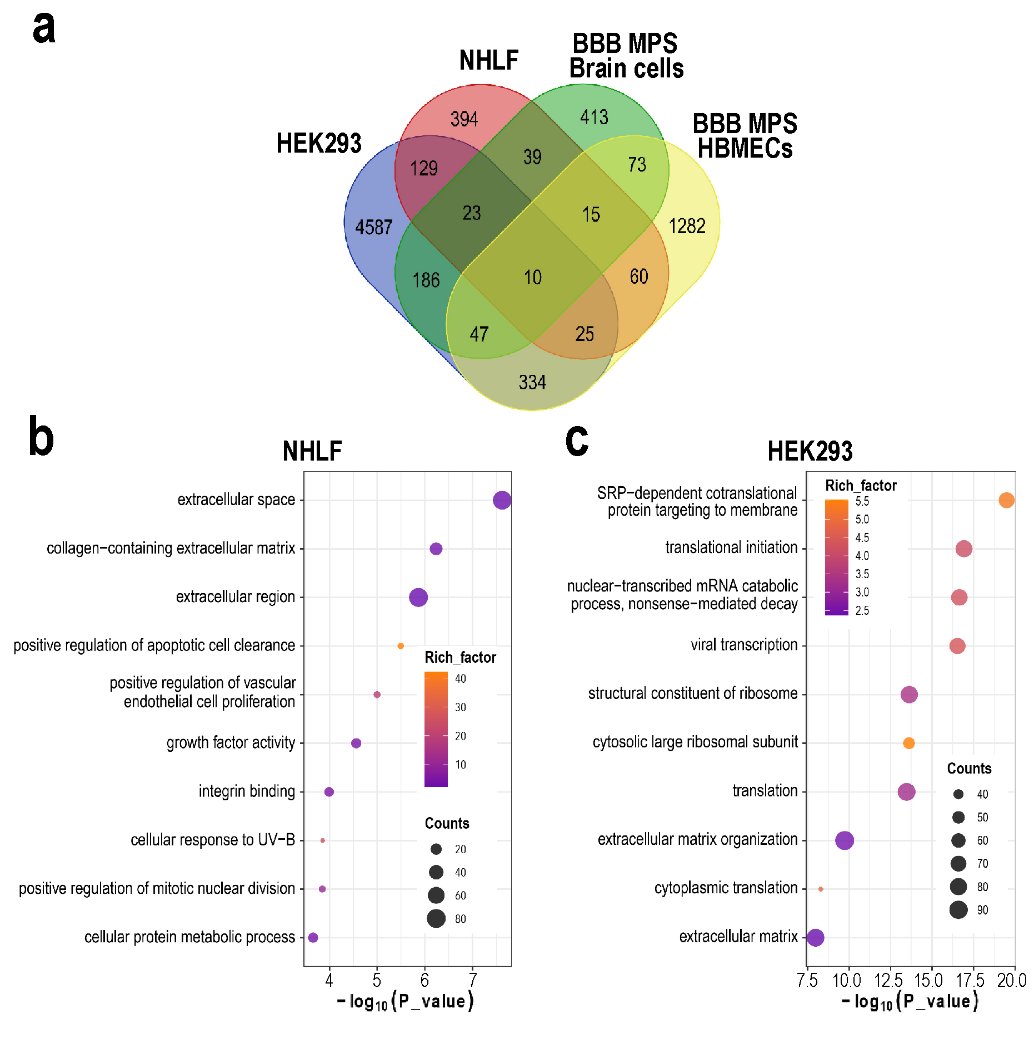


**Supplementary Figure 7. RNA-seq analysis showing transcriptomic responses of NHLF cells and HEK293 cells following γ-ray radiation. a**, Venn diagrams showing overlapping DEGs among NHLF cells, HEK293 cells, brain cells of BBB MPS and HBMECs of BBB MPS following γ-ray radiation. **b**, GO enrichment analysis of DEGs in NHLF cells following γ-ray radiation. **c**, GO enrichment analysis of DEGs in HEK293 cells following γ-ray radiation. Genes differentially expressed with fold changes of > 2.0 and *P* < 0.05 are defined as DEGs. *P* values were calculated using a two-sided, unpaired Student’s t-test with equal variance assumed (*n* = 3).

**
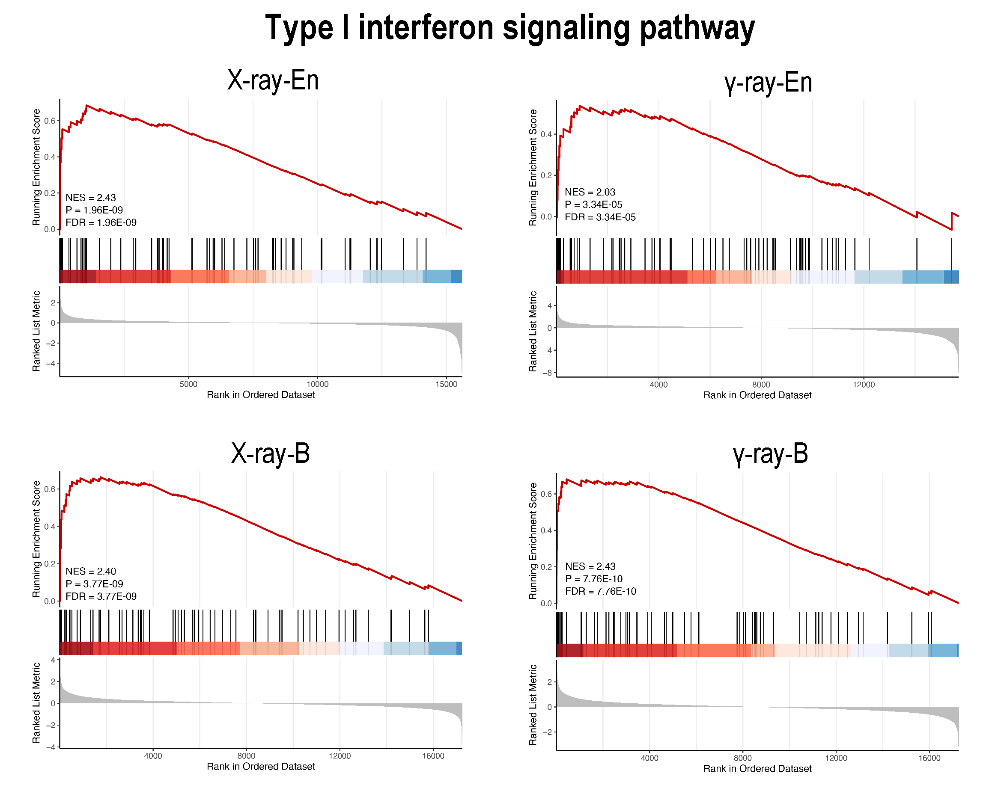
**

**Supplementary Figure 8. GSEA analysis of type I interferon signaling pathway for brain endothelial cells and brain cells on the BBB MPS following radiation exposure. a**, GSEA analysis reveals correlation between radiation exposure and genes involved in type I interferon signaling pathway in brain endothelial cells. **b**, GSEA analysis reveals correlation between radiation exposure and genes involved in type I interferon signaling pathway in brain cells. **a-b**, NES, normalized enrichment score. FDR, false discovery rate. Gene sets were considered significant when *P* < 0.05 and FDR < 0.25.


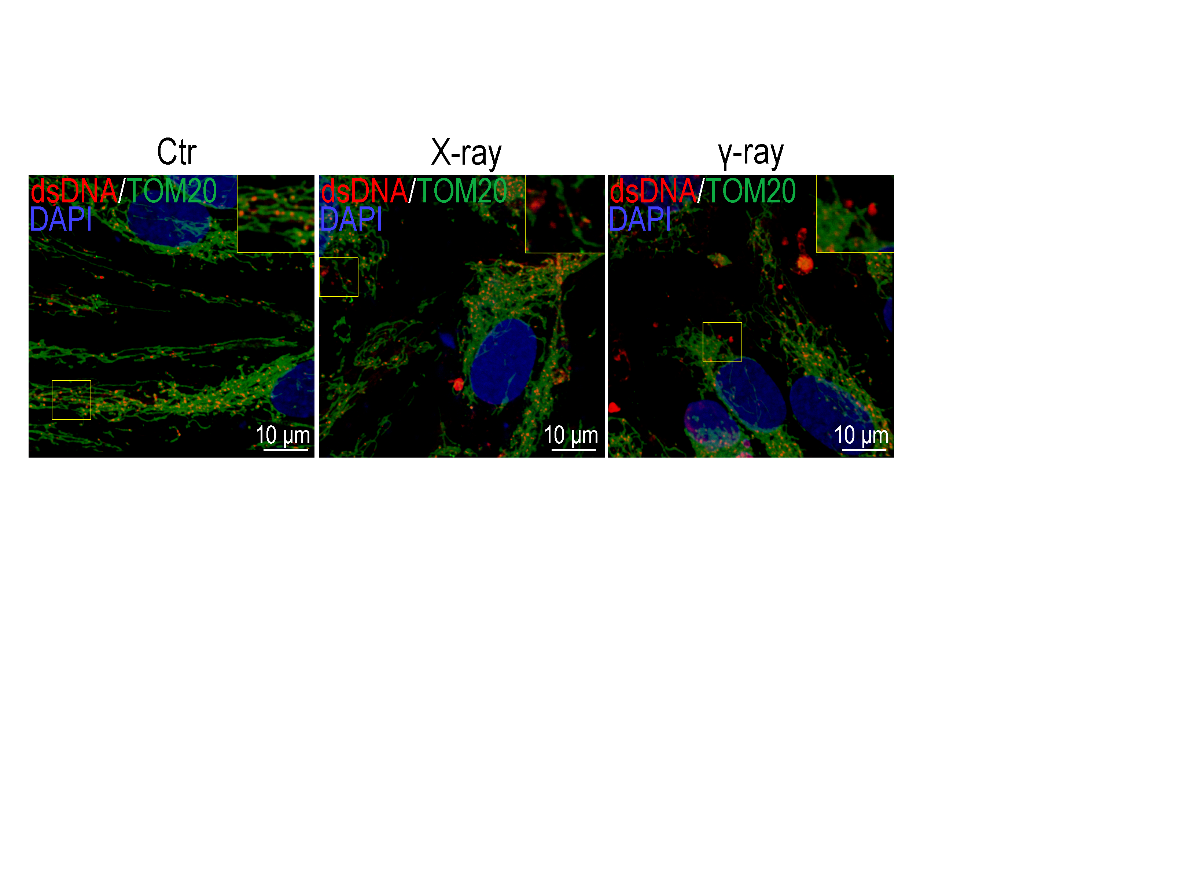


**Supplementary Figure 9. 3D micrographs showing sub-cellular distribution of dsDNA and TOM20 in brain endothelial cells following radiation exposure.** 3D micrographs showing brain endothelial cells immunostained for TOM20 (green) and dsDNA (red) in brain endothelial cells, 4 days after radiation exposure.


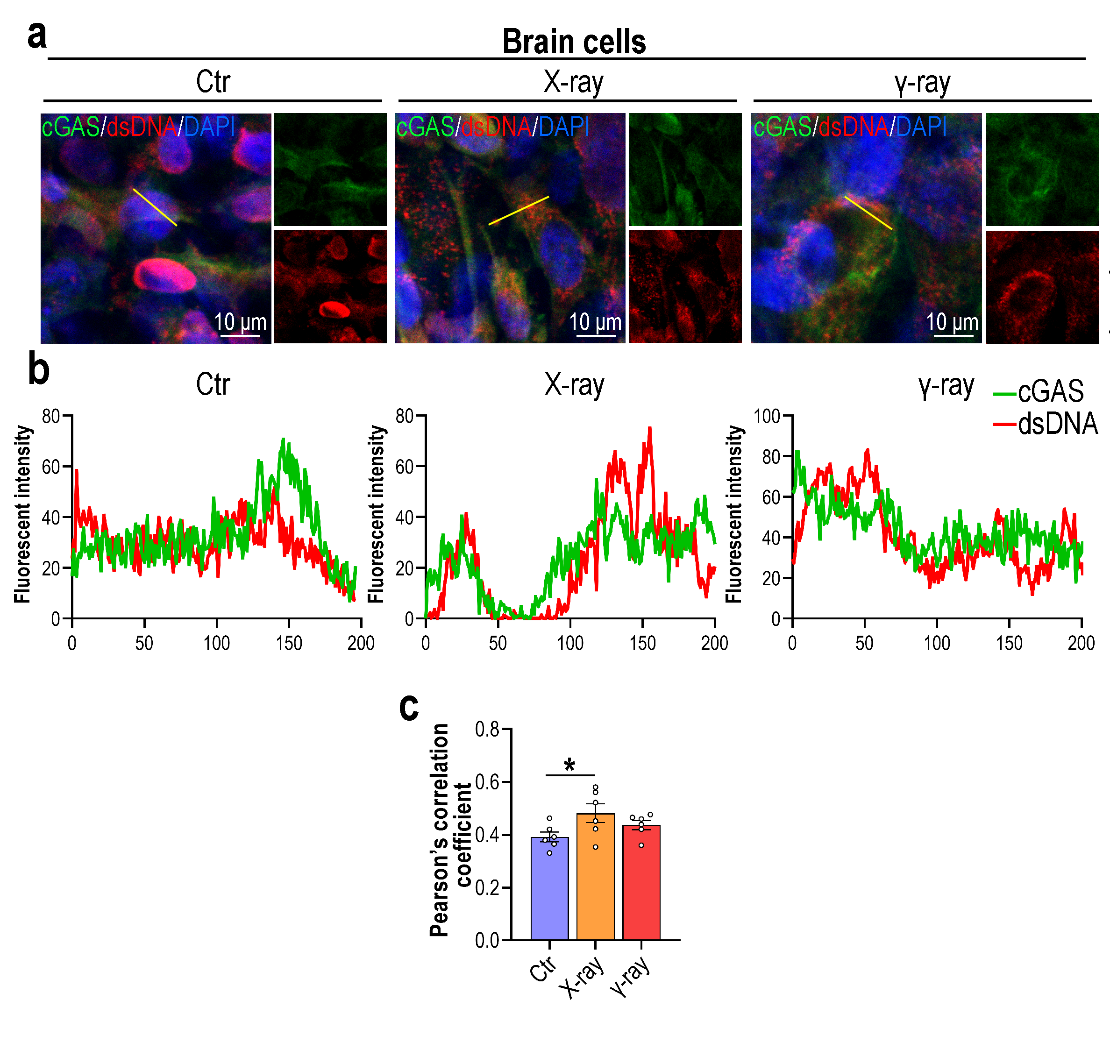


**Supplementary Figure 10. Colocalization analysis of mtDNA and cGAS in brain cells following γ-ray radiation. a**, Confocal micrographs of brain cells immunostained for dsDNA (red) and cGAS (green) at 4 days post-radiation exposure (*n* = 3). **b**, Line-scan analysis of dsDNA (red) and cGAS (green) along the yellow line in **b**. **c**, Quantification of dsDNA (red) and cGAS (green) colocalization using Pearson’s correlation coefficients (*n* = 3). Two fields were quantified per sample. Data are presented as the mean ± SEM (one-way analysis of variance followed by the Bonferroni post-hoc test, *: *P* < 0.05).


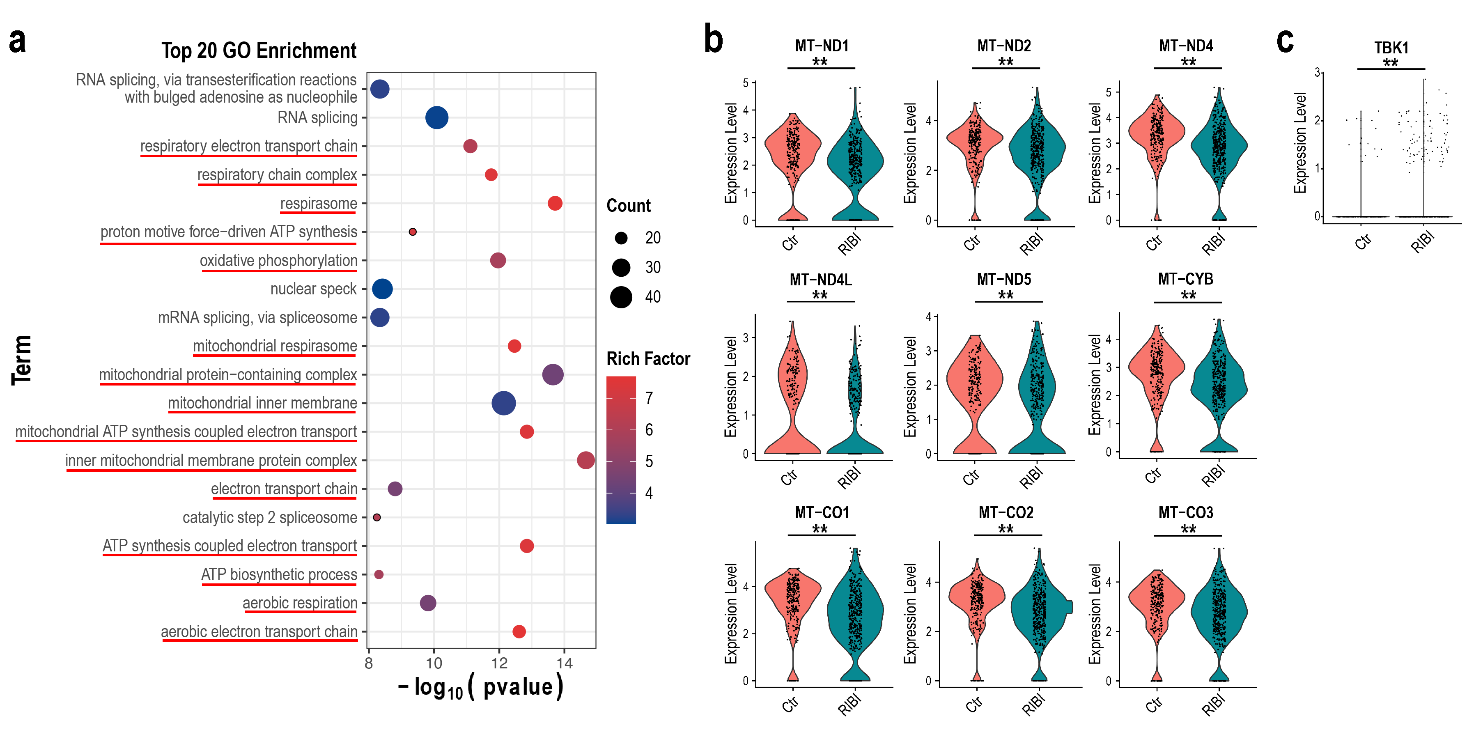


**Supplementary Figure 11. scRNA-seq analysis for brain endothelial cells from RIBI patients. a**, GO enrichment analysis of down-regulated DEGs in brain endothelial cells from RIBI patients’ samples. **b**, Violin plots showing levels of mtDNA-encoded genes in brain endothelial cells from RIBI patients’ samples. **c**, Violin plots showing level of TBK1 in brain endothelial cells from RIBI patients’ samples. The scRNA-seq data was obtained from a study of Tang’s group (Shi et al., 2023).


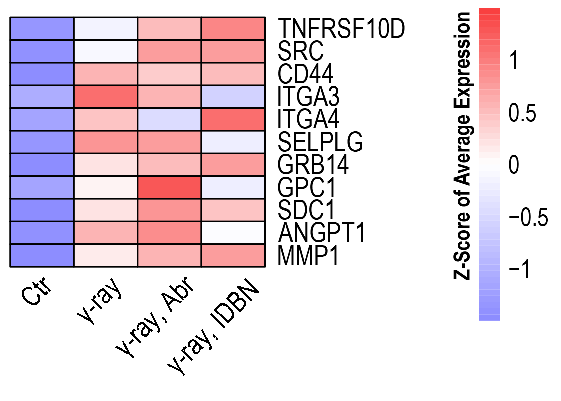


**Supplementary Figure 12. Heatmap showing expression levels of genes related to leukocyte migration in irradiated brain endothelial cells following abrocitinib or idebenone treatment.** Genes differentially expressed with fold changes of > 2.0 and *P* < 0.05 are defined as differentially expressed genes (DEGs). *P* values were calculated using a two-sided, unpaired Student’s t-test with equal variance assumed (*n* = 3).


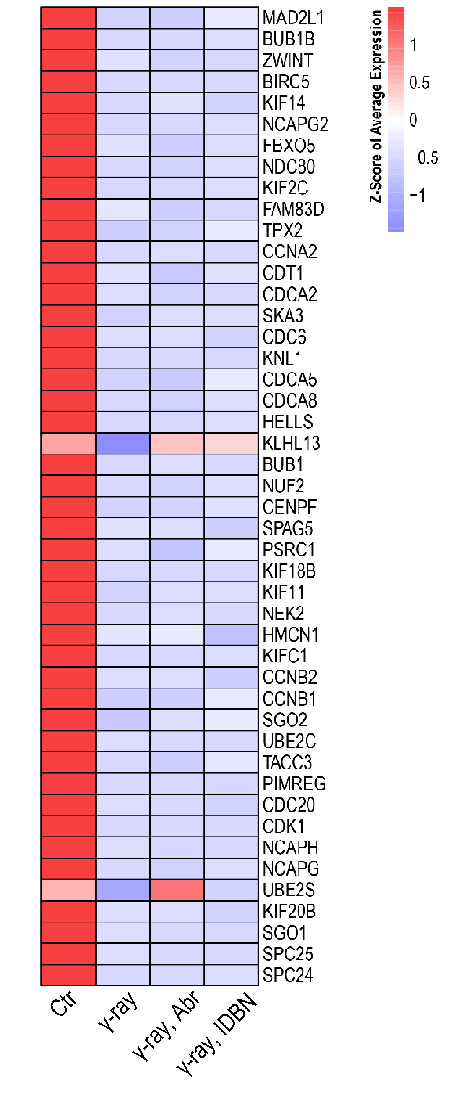


**Supplementary Figure 13. Heatmap showing expression levels of genes related to cell division in irradiated brain endothelial cells following abrocitinib or idebenone treatment.** Genes differentially expressed with fold changes of > 2.0 and *P* < 0.05 are defined as differentially expressed genes (DEGs). *P* values were calculated using a two-sided, unpaired Student’s t-test with equal variance assumed (*n* = 3).

**
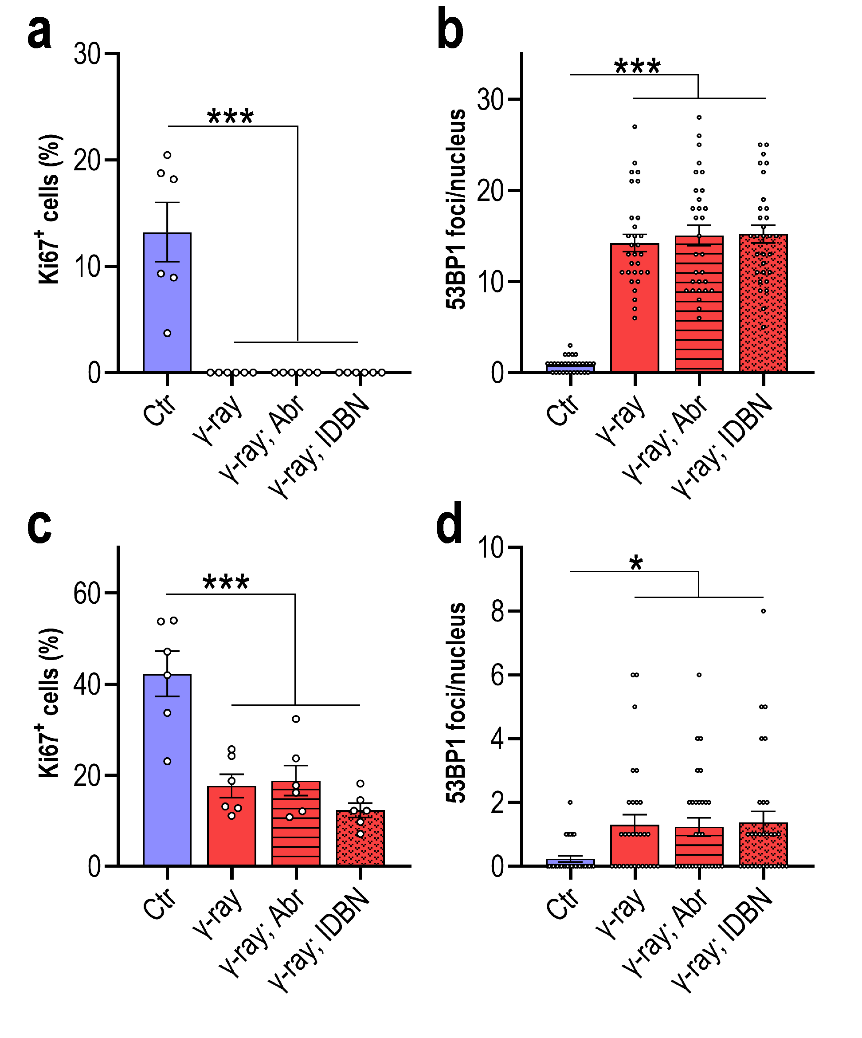
**

**Supplementary Figure 14. Quantification of Ki67+ cells and 53BP1 foci for cells of irradiated BBB MPS following abrocitinib or idebenone treatment. a**, Quantification of Ki67+ cells for brain endothelial cells on irradiated BBB MPS following abrocitinib or idebenone treatment based on **Fig. 6j**. **b**, Quantification of 53BP1 foci per cell for brain endothelial cells on irradiated BBB MPS following abrocitinib or idebenone treatment based on **Fig. 6j**. **c**, Quantification of Ki67+ cells for brain cells on irradiated BBB MPS following abrocitinib or idebenone treatment based on **Fig. 6l**. **d**, Quantification of 53BP1 foci per cell for brain cells on irradiated BBB MPS following abrocitinib or idebenone treatment based on **Fig. 6l**. Data are presented as the mean ± SEM and were analyzed using a one-way analysis of variance (ANOVA) followed by the Bonferroni post hoc test (*: *P* < 0.05; ***: *P* < 0.001).

**Reference:**

Shi, Z., Yu, P., Lin, W.J., Chen, S., Hu, X., Chen, S., Cheng, J., Liu, Q., Yang, Y., Li, S.*, et al.* (2023). Microglia drive transient insult-induced brain injury by chemotactic recruitment of CD8(+) T lymphocytes. Neuron *111*, 696-710 e699.
